# Vitamin B—Can it prevent cognitive decline? A systematic review and meta-analysis

**DOI:** 10.1186/s13643-020-01378-7

**Published:** 2020-05-15

**Authors:** Annika Behrens, Elmar Graessel, Anna Pendergrass, Carolin Donath

**Affiliations:** Center of Health Services Research in Medicine, Department of Psychiatry and Psychotherapy, Friedrich-Alexander-University Erlangen-Nürnberg, Schwabachanlage 6, 91054 Erlangen, Germany

**Keywords:** Prevention, Dementia[Mesh], Cognition[Mesh], Vitamin B_6_[Mesh], Vitamin B_12_[Mesh], Folic Acid[Mesh]

## Abstract

**Background:**

Development of cognitive decline represents substantial issues in today’s society, steadily gaining importance with increasing life expectancy. One potential approach to preventing cognitive decline is to lower homocysteine by administering vitamin B. In this systematic review and meta-analysis, we address this topic and investigate whether oral supplementation of vitamin B can successfully prevent cognitive decline in cognitively unimpaired individuals.

**Methods:**

A computerized systematic literature search was conducted using the electronic databases PubMed, Embase, and the Cochrane Library. Eligibility criteria included oral supplementation with vitamin B (B_1_, B_6_, folic acid, and B_12_) and the absence of cognitive impairment. A meta-analysis was conducted with “global cognition” as the primary outcome of this review. Secondary outcomes were changes in cognitive function in other cognitive domains reported in the included studies. Risk of bias was assessed according to the Cochrane Risk of Bias tool and the GRADE approach to establish the overall certainty of the evidence.

**Results:**

The meta-analysis did not yield a significant overall effect of supplementation with vitamin B on cognitive function (*Z* = 0.87; *p* = 0.39; SMD, 0.02; 95% CI, − 0.034, 0.08). A sensitivity analysis focusing on specific risk factors did not alter this result. Some studies reported isolated significant effects of the intervention on secondary outcomes. However, these findings were outnumbered by the number of cognitive tests that did not yield significant effects.

**Discussion:**

We found no overall evidence that oral vitamin B supplementation prevented cognitive decline. The isolated significant effects that were reported could be attributed to methodological issues. The results of this review do not provide evidence that population groups with certain risk factors would profit more from the intervention than others. Our findings do not apply to forms of administration other than oral supplementation nor do they offer information regarding the treatment of cognitively impaired individuals via the administration of vitamin B.

**Systematic review registration:**

PROSPERO CRD42017071692

## Background

Cognitive decline, including forms of dementia such as Alzheimer’s disease, affected an estimated 35.6 million people worldwide in 2009, with an anticipated increase to 66 million by 2030 and 115 million by 2050 [1]. Considering the consistent increase in life expectancy and health care quality, cognitive impairment is becoming an increasingly substantial issue for today’s society [1–4]. The pathogenesis and gradual development of cognitive decline is multi-factorial and thereby various approaches may be promising for prevention and treatment [5, 6]. Mild cognitive impairment and dementia (including Alzheimer’s disease) are only two examples of various reasons for cognitive decline. Cognitive impairment and its preliminary stages affect members of society with respect to various aspects of their day-to-day lives. It is not only the individuals experiencing cognitive decline who are affected, but also their relatives and caregivers. Moreover, the costs resulting from cognitive impairment have an immense economic impact on health care systems [7–12].

Prevention has therefore become a much studied topic in the field of cognitive impairment research, and multiple approaches have been adopted, including life style modification, dietary changes, and nutrient supplementation [13]. Elevated homocysteine blood levels have been identified as one potentially modifiable risk factor for the development of cognitive impairment [14–17]. Homocysteine is an amino acid produced in the process of metabolizing the essential amino acid methionine. Methionine is crucial for a large number of biochemical processes. Via the methionine-homocysteine cycle, homocysteine is either re-metabolized into methionine or converted into the amino acids cysteine and taurine. Folate and vitamin B_12_ are essential for the re-methylation from homocysteine to methionine whereas vitamin B_6_ catalyzes the conversion of homocysteine into other amino acids [6, 18]. Elevated plasma homocysteine indicates failure in the methionine-homocysteine cycle and can result in far-reaching health impairments that affect all life stages ranging from prenatal development to late adulthood [17]. As mentioned above, one condition that can result from pathologically high plasma homocysteine is cognitive decline. High homocysteine is associated with several neurodegenerative disorders, including general cognitive impairment, mild cognitive impairment, Alzheimer’s disease, and dementia [15, 18]. It is toxic to vascular endothelial and neuronal cells and thereby contributes to brain atrophy and the degeneration of neurons [6, 19].

A possible cause for a breakdown in the methionine-homocysteine cycle, characterized by elevated homocysteine levels, is a deficiency in vital nutrients (i.e., in our case, vitamin B). Because representatives of the vitamin B complex are so closely linked to the physiological metabolism of homocysteine, a lack of these would severely impact the recycling of homocysteine [17, 20]. Thus, the idea that supplementing vitamin B could forestall cognitive decline has emerged and became a much studied topic. The question of whether a lowering of homocysteine via vitamin B supplementation can successfully prevent cognitive impairment has been addressed in various studies. In addition to the homocysteine-linked vitamins folate, B_6_, and B_12_, vitamin B_1_ has also been associated with cognitive impairment and the development of neurodegeneration [21, 22]. This representative of the vitamin B complex is crucial for retrieving energy from carbohydrates and supports the functioning of the human nervous system [6].

Previous reviews and meta-analyses have reported inconsistent results and conclusions and have often called for further high-quality, long-term trials on this topic [23–29]. However, none of these conclusions should be embraced without caution and critical questioning. For instance, some articles have included both prevention and treatment, thus mixing two separate topics in the same analysis [23, 25]. Extraction of specific information on vitamin B supplementation in the cognitively unimpaired individuals from the text of such articles is therefore impeded. One meta-analysis addressed the same primary outcome that we did, that is, global cognition [24]. However, we included several studies that were not assessed in the older meta-analysis, thus justifying ours. No previous review or meta-analysis has considered categorizing cognitively unimpaired participants via their risk factors for developing cognitive impairment.

In this systematic review, on the basis of the available evidence, we aimed to draw a definitive conclusion on whether the oral supplementation of vitamin B_1_, B_6_, B_12_, and folic acid (in the following referred to as “vitamin B”) could be used to prevent cognitive decline in cognitively unimpaired individuals. Therefore, we focused exclusively on trials that specifically excluded participants with cognitive impairment such as MCI or dementia, thereby differentiating our concept from older reviews that included both prevention and treatment trials. We also took into consideration the fact that, despite the absence of cognitive impairment, different risk factors for developing cognitive decline may be present for the participants. A potential beneficial effect of vitamin B on cognition would most likely be observed for certain population groups [29]. Thus, we attempted to cluster the results of this review into population groups—healthy individuals versus individuals with pre-existing risk factors such as, elevated homocysteine levels, vitamin B deficiency, and other pre-existing conditions. This approach, to our knowledge, has not been implemented in a systematic review or meta-analysis before.

To achieve this goal with a systematic literature review, we assessed (placebo-) controlled trials that assessed the oral supplementation of vitamin B_1_, B_6_, B_12_, and folic acid single or in combinations (in the following referred to as “vitamin B”) in cognitively unimpaired individuals. We explicitly excluded any studies in which participants had any form of pre-existing cognitive impairment or used any form of administration of vitamin B other than oral supplementation. We examined post-intervention differences in cognitive function between the intervention and the placebo groups. We conducted a meta-analysis to assess the primary outcome of this review.

## Methods

### Eligibility criteria

This review was submitted to the PROSPERO register and was filed under the following registration number: CRD42017071692. We conducted this review according to the PRISMA statement and checklist and therefore followed PRISMA’s recommendation to define our eligibility criteria using the PICOS approach. The PRISMA statement is a tool to improve reporting in systematic reviews, particularly for those assessing randomized controlled trials. It consists of a checklist comprising the sections title, abstract, introduction, methods, results, discussion, and funding of the systematic review as well as a flow chart to visualize and document the study selection process [30, 31]. The PRISMA checklist is attached as Additional file 1.

Detailed eligibility criteria (PICOS) as defined before we conducted the initial data base search are presented in Additional file 2. We searched for controlled trials that assessed an oral supplementation of the vitamin B representatives B_1_, B_6_, B_12_, and folic acid in cognitively unimpaired individuals. We excluded pre-existing mental disorders. Other pre-existing conditions were not excluded. Outcomes of interest were changes in cognitive performance from baseline to the last follow-up available for different cognitive domains. We defined global cognition as the primary outcome and summarized other reported cognitive domains under the secondary outcomes. Studies that did not provide information on changes of cognitive function were excluded. We set restrictions for age, focusing on adults and therefore excluding children and adolescents as well as pregnant women and newborns. No restrictions were set concerning race, origin, year of publication, language, or length of follow-up. Only published, peer-reviewed journal articles were considered eligible.

### Data synthesis

We conducted a computerized literature search using the electronic databases PubMed, Embase (Excerpta Medica Database), and the Cochrane Library. All database searches were last updated on October 9, 2019. We identified one additional record through snowballing. Table 1 shows the search strategy used for PubMed. No restrictions other than the search terms were set.

**Table 1:** Search strategy used in the PubMed database search

	(“Vitamin B Complex”[Mesh] OR “Thiamine”[Mesh] OR “Vitamin B6”[Mesh] OR “Folic Acid”[Mesh] OR “Vitamin B12”[Mesh] OR “Vitamin B”[Title/Abstract] OR “Thiamine”[Title/Abstract] OR “Vitamin B1”[Title/Abstract] OR “Pyridoxine”[Title/Abstract] OR “Vitamin B6”[Title/Abstract] OR “Folic Acid”[Title/Abstract] OR “Folate”[Title/Abstract] OR “Vitamin B9”[Title/Abstract] OR “Cobalamine”[Title/Abstract] OR “Vitamin B12”[Title/Abstract] OR “Homocysteine”[Title/Abstract] OR “Hyperhomocysteinemia”[Title/Abstract])
AND	(“Humans”[Mesh])
AND	(“Cohort”[Title/Abstract] OR “Follow-Up”[Title/Abstract] OR “Longitudinal”[Title/Abstract] OR “Prospective”[Title/Abstract] OR “Retrospective”[Title/Abstract] OR "Study"[Title/Abstract] OR "Trial"[Title/Abstract]) AND “controlled”[Title/Abstract])
AND	(“Cognitive”[Title/Abstract] OR “Cognition”[Title/Abstract])

**Fig. 1:**
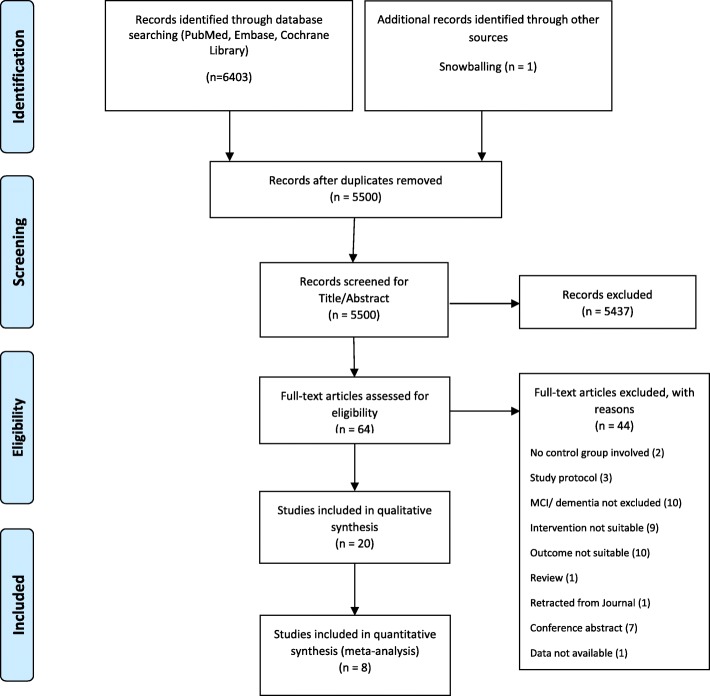
Study selection process. *n* = number of records

Our study selection process is shown in Fig. 1, depicting the PRISMA flowchart for the study identification and selection process [30, 31]. We initially retrieved 6404 records. After we removed duplicates, 5500 records were left and were then screened for title and abstract according to the criteria listed in Additional File 2. During this part of the screening process, we excluded 5437 articles that did not meet the eligibility criteria. Afterwards, we assessed the full texts of 63 records for further eligibility and included 20 records in this review. Reasons for the exclusion of full-text articles are stated within Fig. 1. Eight of the 20 records included in the qualitative synthesis addressed the same cognitive outcome construct and were therefore included in the meta-analysis. Database literature searches were conducted and updated by the lead author AB. Record screening for title and abstract as well as the full-text assessments were each conducted independently by the lead author AB and an additional reviewer CV. Dissents were solved by discussion between AB and CV and, if still existent afterwards, by consulting one of the co-authors (EG, CD, or AP) as a third reviewer for the respective articles.

To determine further eligibility, the records considered for full-text assessment were read completely and logged into a standardized, pre-piloted form the authors EG and AP developed in-house. The categories in this form were as follows: author, country, year, study type/design, study population, number of participants, details about the intervention and control conditions, outcomes, times of measurement, results, form of prevention, any particularities we considered worth mentioning (e.g., the study was performed as part of another, large-scale trial), and the duration of the trial. The eligibility criteria adapted for the assessments of the full text are summarized in Table 2. If we needed to obtain more information about particular records, we contacted the affiliated authors. Specifically, this was used to obtain missing outcome data.

**Table 2:** PICOS eligibility criteria as adapted for full-text assessment

	Included in review	Excluded from review
Patient population	Healthy individuals Pre-existing conditions other than stated on the right	Any form of cognitive impairment Pre-existing mental disorders Children and adolescents Pregnant women
Intervention	Oral supplementation with vitamin B • B_1_/B_6_/folic acid/B_12_ • Single or in different combinations • Combined with other micro-nutrients	Additional interventions other than stated on the left Use of vitamin B not explicitly stated
Comparison	Control group (placebo)	Any other
Outcomes	Global cognition Change in cognitive performance from baseline to the follow-up last available	Non-cognitive outcome measures Studies lacking data on change of cognitive measures
Study design	RCTs (randomized controlled trials) Controlled studies	Reviews Commentaries Non-controlled studies (pre-post design)

### Risk of bias

We employed the Cochrane Risk of Bias Tool to assess the risk of bias at the outcome level in individual studies concerning six mandatory categories [32]. The seventh category “other bias” from the Risk of Bias Tool (not mandatory) was used only to report any particularities found in studies that were not covered by the other categories. If no additional particularities were found, this category was given a “low risk” rating. The information we retrieved was used to interpret the results and to include the results in a sensitivity analysis. Details on the risk of bias evaluation are presented separately for all included studies for those included in the meta-analysis. For further evaluation of the risk of bias on the outcome level across studies, a GRADE evaluation was conducted on our primary as well as on our secondary analyses [33–35].

### Data analysis

We defined global cognition as the primary outcome of this review. The primary outcome measure was thus the standardized mean difference in global cognition between the intervention and control groups at follow-up. We retrieved all studies that included either the MMSE (Mini-Mental Status Examination) or the TICS-m (Telephone Interview for Cognitive Status, modified version) and incorporated them into the meta-analysis. There is evidence that MMSE and TICS scores can be linked directly (i.e., they measure the same construct), thereby justifying the integration of these studies into one meta-analysis [36–38]. Secondary outcomes presented in the main review consist of the single cognitive domains processing speed, memory, verbal ability, executive function, and attention, while information on other cognitive domains is made available in the Additional files 3 and 5.

We present the results according to population groups. To display a meaningful synthesis of our results, we categorized the populations from the studies into four population groups: healthy individuals [39–46], those with vitamin B deficiency [47, 48], those with elevated homocysteine levels [49–51], and those with other risk factors for cognitive decline [52–58] (see Table 4). This categorization can be justified by taking into consideration the fact that each of the groups (except “healthy individuals”) represents a risk factor for developing cognitive impairment. The metabolic functions of elevated homocysteine levels and low vitamin B levels are related and can both be linked to cognitive impairment dependently and independently [6, 14–16, 20, 59–62]. The studies that were categorized into the population group representing other risk factors included individuals with cardiovascular diseases (i.e., hypertensive disease, ischemic vascular disease, and a history of transient ischemic attacks, ischemic stroke, or myocardial infarction), chronic kidney disease, and diabetes [63–71].

The meta-analysis (quantitative synthesis) was conducted using the Cochrane Review Manager 5, where data for the same outcome but measured with different scales can be integrated and standardized [72].

In accordance with the Cochrane Risk of Bias evaluation recommendations, we conducted a post hoc sensitivity analysis through which we excluded studies to which we attributed a high risk of bias. We defined an overall high risk of bias as a high-risk rating in two or more of seven categories provided by the Cochrane Risk of Bias tool.

The main meta-analysis was carried out independently from the defined population groups. We applied a corresponding post hoc subgroup analysis in order to foster the homogeneity of the population groups in the included studies.

To provide an organized overview of the reported effects regarding the secondary outcomes, we decided to structure the qualitative synthesis according to the defined population groups. For each of the four population groups, we developed a table comprising the assessed single cognitive domains and their effects (included as Additional file 4). To ensure clarity and economy in the main review, we decided to include an overview table comprising the categories that showed significant results (Table 5).

We post hoc computed an additional analysis for potential intervention risk detection. There, we assessed effects that favored placebo over the administration of vitamin B. It is enclosed as Additional file 5.

## Results

### Study selection

Through our database research, we identified a total of 6404 records; after duplicates were removed, 5500 records were screened for title and abstract. After the first screening process, 63 articles were read in full and assessed for further eligibility. Twenty studies were included in the systematic review and underwent the qualitative synthesis, and 8 of these were deemed appropriate for the meta-analysis [50–56, 58]. For a detailed flow chart of the study selection process including reasons for exclusion, see Fig. 1.

Although we did not explicitly exclude non-RCTs, all studies that met the inclusion criteria were randomized, placebo-controlled trials.

### Study characteristics

Overall, 12,697 participants of interest for this review were included in the 20 studies, ranging from 23 to 2919 participants per study. The patient population consisted solely of outpatients who were orally supplementing their diets vitamin B. In the majority of the studies, elderly participants with an age of 60 years or older were assessed [41–43, 45–48, 50, 51, 54, 57, 58]. Three studies included middle-aged to elderly individuals (i.e., 45–80 years, > 40 years, and 50–70 years) [49, 52, 56]. Four studies also included young adults (i.e., 20–49 years, 18–86 years, 20–92 years, and over the age of 21 years) [39, 40, 44, 53]. One trial did not set any age-related restrictions [55]. While most studies included both male and female participants, 3 studies included only women [39, 46, 56] and one study assessed only men [54]. Four trials investigated the effects of a multivitamin formula on cognitive function but explicitly included vitamin B in their analysis and were therefore included [40, 41, 44, 46].

The characteristics of the studies and interventions are presented in Table 3.

**Table 3:** Overview: trial and intervention details

Trial characteristics				Intervention			
Study	Country	Design	***n*** = participants in eligible (subgroup) cognitive testing*	B vitamins supplemented	Dosage	Duration of treatment	(Last) follow-up
Andreeva et al. [52]^a^	France	RCT	871	Folate, vitamin B_6_, vitamin B_12_	Daily: Folate 0.56 mg B_6_ 3 mg B_12_ 0.02 mg	4 years	End of treatment
Brady et al. [53]	The USA	RCT	659	Folate, vitamin B_6_, vitamin B_12_	Daily: Folic acid 40 mg B_6_ 100 mg B_12_ 2 mg	5 years	After 4 and 5 years of treatment
Bryan et al. [39]	Australia	RCT	211	Folate, B_6_, B_12_	Daily: Folate 750 μg Or daily: B_6_ 20 mg Or daily: B_12_ 15 μg	5 weeks	End of treatment
Chan et al. [40]	The USA	RCT	115	Nutriceutical formula also containing folic acid and B_12_	Daily: Folic acid 400 μg, B_12_ 6 μg	3 months	End of treatment
Cockle et al. [41]	The UK	RCT	139	Multivitamin supplement also containing folic acid, B_6_, B_12_	Daily: Folic acid 600 mg, B_6_ 22 mg, B_12_ 0.03 mg	24 weeks	End of treatment
Dangour et al. [47]	The UK	RCT	201	B_12_	Daily: B_12_ 1 mg	1 year	End of treatment
Durga et al. [49]	Netherlands	RCT	818	Folic acid	Daily: Folic acid 800 μg	3 years	End of treatment
Eussen et al. [48]	Netherlands	RCT	195	Vitamin B_12_ or vitamin B_12_ + folate	Daily: B_12_ 1000 μg Or daily: B_12_ 1000 μg Folic acid 400 μg	24 weeks	End of treatment
Ford et al. [54]	Australia	RCT	299	Folic acid, vitamin B_6_, vitamin B_12_	Daily: Folic acid 2 mg B_6_ 25 mg B_12_ 400 μg	2 years	End of treatment
Hankey et al. [55]^a^	Netherlands	RCT	2214	Folic acid, vitamin B_6_, vitamin B_12_	Daily: Folic acid 2.5 mg B_6_ 25 mg B_12_ 500 μg	3.4 years	End of treatment
Kang et al. [56]	The USA	RCT	2009	Folic acid, vitamin B_6_, vitamin B_12_	Daily: Folic acid 2.5 mg B_6_ 25 mg B_12_ 500 μg	6.6 years	End of treatment
Kwok et al. [57]	China	RCT	271	Vitamin B_12_	Daily: B_12_ 1000 μg	27 months	End of treatment
Lewerin et al. [42]	Sweden	RCT	195	Folate, vitamin B_6_, vitamin B_12_	Daily: Folate 0.8 mg B_6_ 3 mg B_12_ 0.5 mg	4 months	End of treatment
McMahon et al. [50]	New Zealand	RCT	276	Folate, vitamin B_6_, vitamin B_12_	Daily: Folate 1000 μg B_6_ 10 mg B_12_ 500 μg	2 years	End of treatment
Pathansali et al. [43]	The UK	RCT	24	Folic acid	Daily: Folic acid 5 mg	4 weeks	End of treatment
Pipingas et al. [44]	Australia	RCT	138	Multivitamin formula also containing folic acid, vitamin B_6_, and vitamin B_12_	Daily: Folic acid 500 μg B_6_ 41.14 mg B_12_ 50 μg	16 weeks	End of treatment
Stott et al. [58]^a^	The UK	RCT	23	Folic acid, vitamin B_6_, and vitamin B_12_ as one of various combinations	Daily: Folic acid 2.5 mg B_6_ 25 mg B_12_ 400 μg	12 weeks	After 1 year
Van der Zwaluw et al. [51]	Netherlands	RCT	2919	Folic acid, vitamin B_12_	Daily: Folic acid 400 μg B_12_ 500 μg	2 years	End of treatment
Walker et al. [45]	Australia	RCT	900	Folic acid, vitamin B_12_	Daily: Folic acid 400 μg B_12_ 100 μg	2 years	End of treatment
Wolters et al. [46]	Germany	RCT	220	Multivitamin capsules also containing folic acid, vitamin B_6_, and vitamin B_12_	Daily: Folic acid 400 μg B_6_ 3.4 mg B_12_ 9 μg	6 months	End of treatment

^a^For studies that met our inclusion criteria only in a specific subgroup, *n* indicates the number of participants in said subgroup

We present the results according to population groups. To display a meaningful synthesis of our results, we categorized the populations from all studies into four groups: healthy individuals, those with elevated homocysteine levels and those with low vitamin B levels as risk factors for cognitive impairment, and those with other risk factors for cognitive decline (i.e., cardiovascular diseases, chronic kidney disease, and diabetes). An overview of the population groups is shown in Table 4. The population group of healthy individuals comprised 8 studies [39–46]. Individuals with vitamin B deficiency were assessed in 2 trials [47, 48]. The population group with elevated homocysteine levels consisted of 3 studies [49–51]. Seven studies were categorized into the population group with other risk factors [52–58].

**Table 4:**
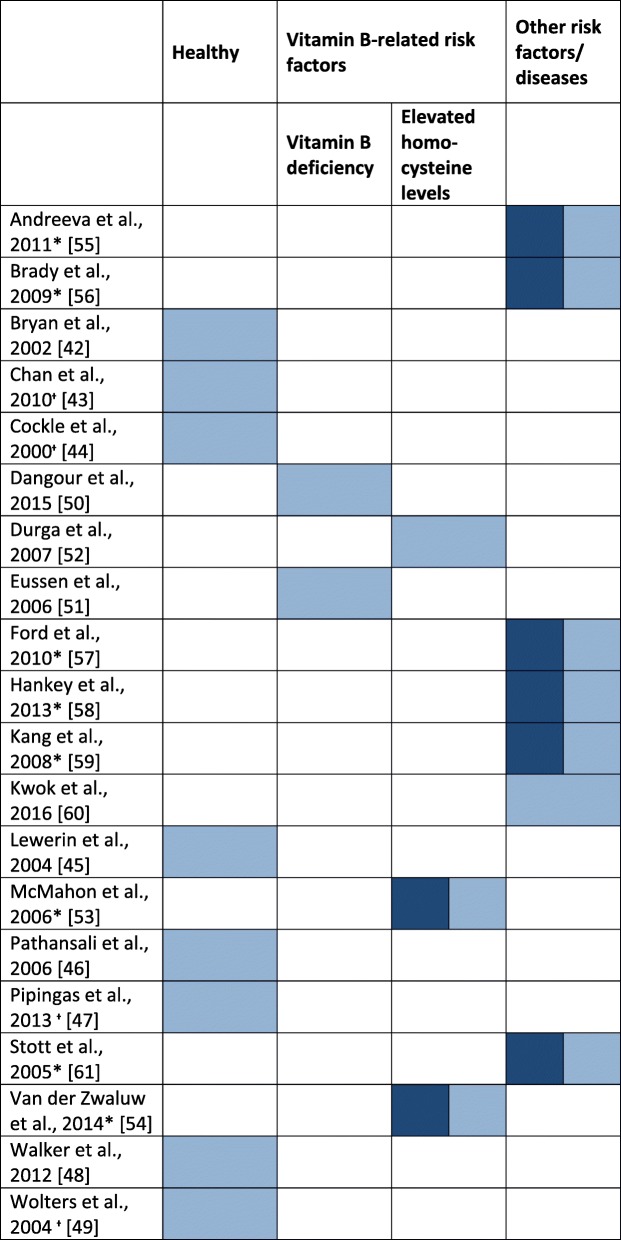
Assignment of the in the systematic review included studies to the four population groups [39, 40, 46, 48, 49, 54]

Dark blue = primary outcome; light blue = secondary outcomes

*Included in meta-analysis

^+^Use of multivitamin formula

### Risk of bias within studies

**Fig. 2:**
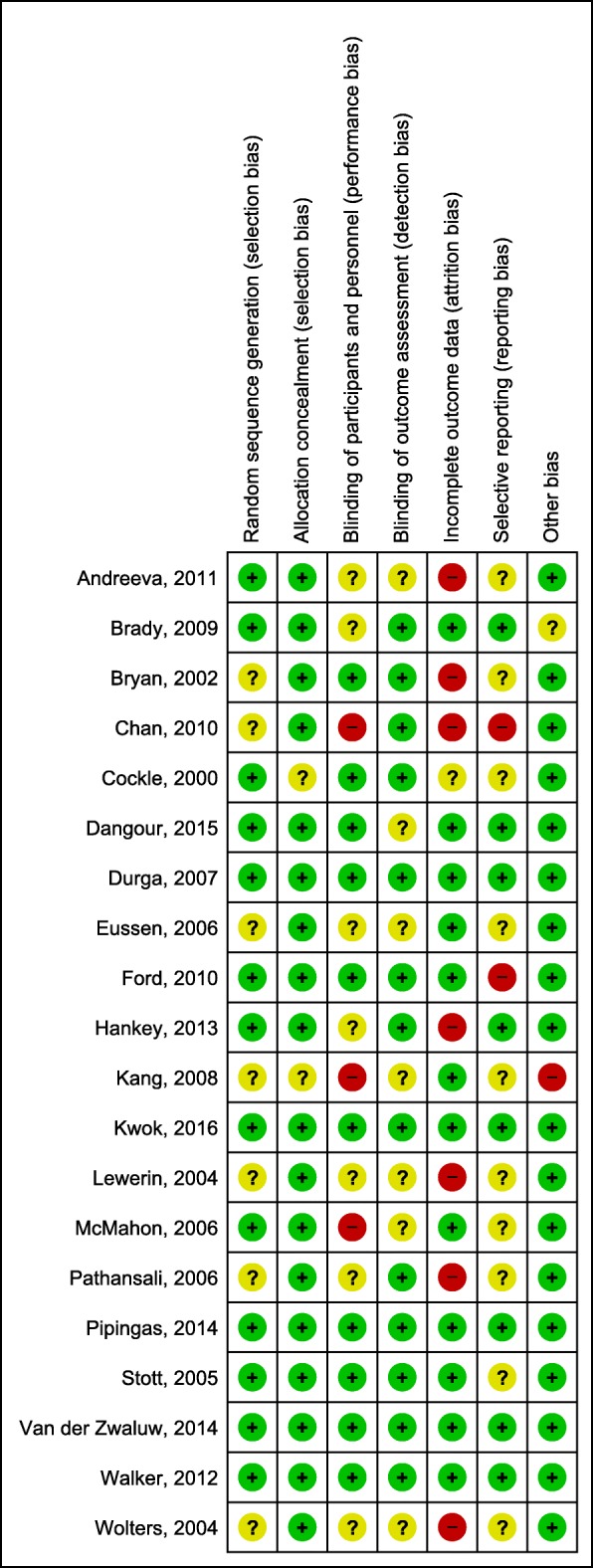
Risk of bias overall summary. Green = low risk of bias; yellow = unclear risk of bias; red = high risk of bias

**Fig. 3:**
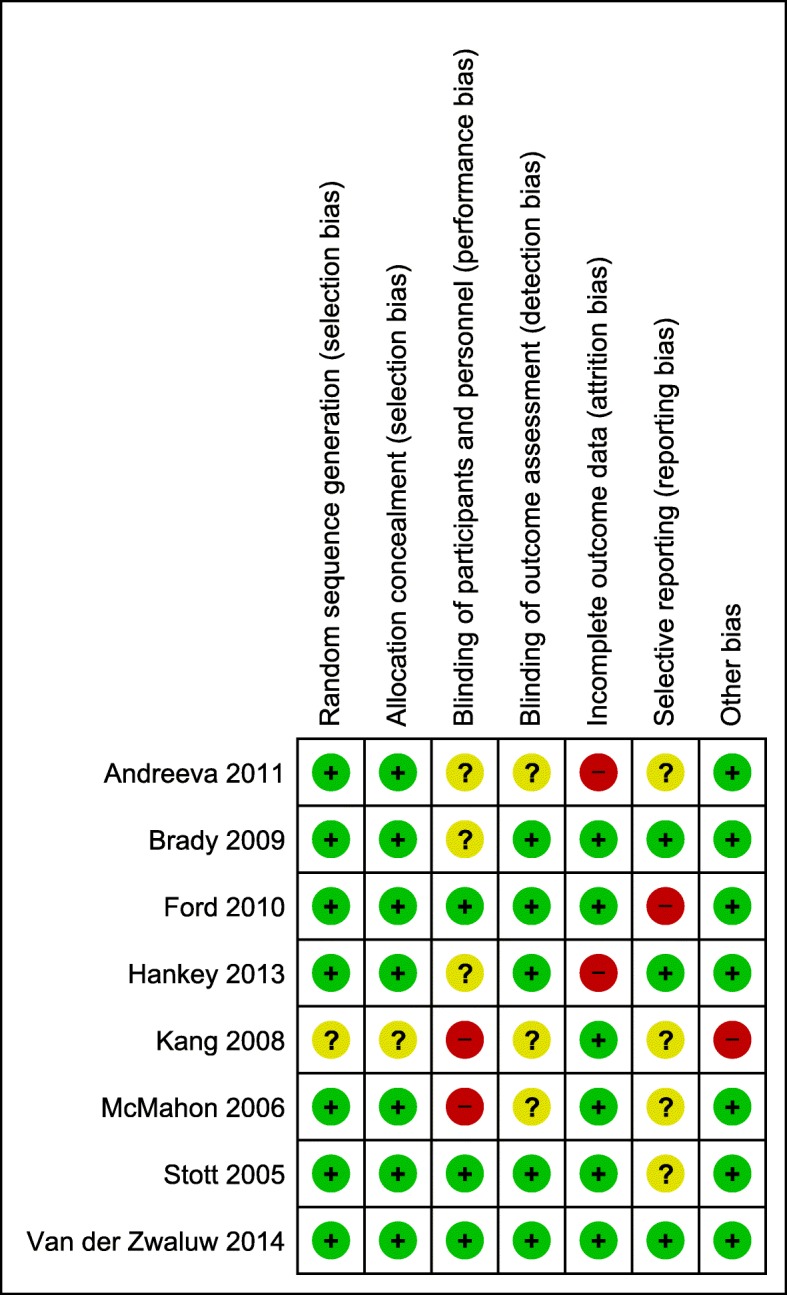
Risk of bias summary of studies included in the meta-analysis. Green = low risk of bias; yellow = unclear risk of bias; red = high risk of bias

We assessed all 20 studies individually via the Cochrane Risk of Bias tool. Details on the risk of bias evaluation are presented separately for all included studies and for those included in the meta-analysis. Figure 2 shows the risk of bias summary chart for all 20 studies, whereas Fig. 3 includes only those incorporated into our meta-analysis. As Fig. 2 shows, most studies had a low risk of selection bias and a low or unclear risk of detection bias. Three studies were given a high-risk rating for performance bias [40, 50, 56], 2 studies for reporting bias [40, 54], and one study for other bias [56]. Attrition bias was the most common category to receive a high-risk rating, affecting 7 out of the 20 studies, 2 of which were included in the meta-analysis (see Fig. 3) [39, 40, 42, 43, 46, 52, 55]. The overall risk of bias in individual studies was mostly low to moderate. Two studies were attributed an overall high risk of bias; one of them was featured in the primary outcome and was thus excluded in the second step of the meta-analysis [40, 56].

### Synthesis of results

#### Primary outcome

**Fig. 4:**
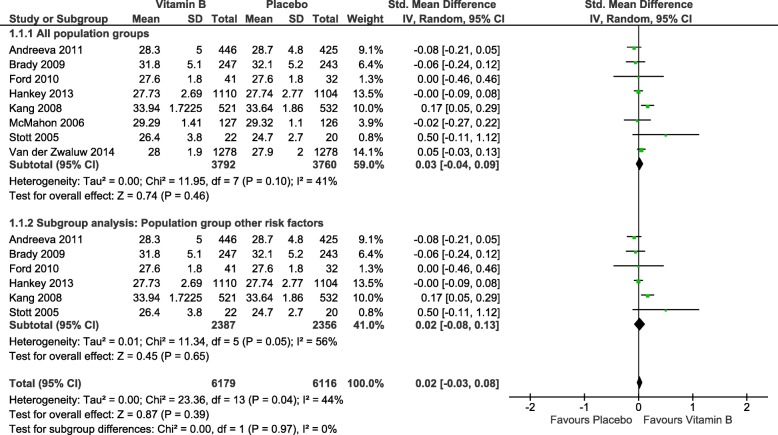
Forest plot: global cognition—meta-analysis. SD, standard deviation; CI, confidence interval

**Fig. 5:**
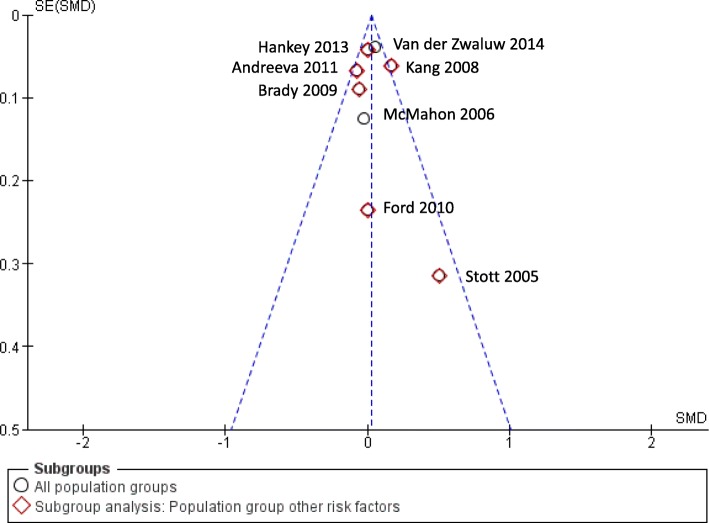
Funnel plot: global cognition—meta-analysis

We defined global cognition as the primary outcome of this review. Global cognition can be measured with different assessment tools. The tools that were most commonly used in the included studies were the MMSE (Mini-Mental Status Examination) and the TICS-m (Telephone Interview for Cognitive Status, modified version) [36, 73–75]. Thus, we decided to retrieve all studies that included either the MMSE or the TICS-m as outcome measure and incorporated them into the meta-analysis. The meta-analysis (quantitative synthesis) was conducted using the Cochrane Review Manager 5, where data for the same outcome but measured with different scales can be integrated and standardized [72]. We selected the continuous data type and used the standardized mean difference, random effects model (CI (confidence interval) 95%). PRISMA states that the standardized difference in means is the appropriate measure for data drawn from different scales, as done in our meta-analysis. We used the random effects model because the data is heterogeneous as there are differences throughout the studies regarding patient populations, intervention details, and time of follow-up. Heterogeneity was determined using *I*^2^. Outcome reporting across studies included in our meta-analysis consisted of baseline and direct post-intervention assessment data for most of the studies. One trial was implemented as a sub-study for cognitive testing, 3 years after treatment began in accordance with the parent study [53]. Thus, the provided cognitive baseline data does not necessarily represent the cognitive function of the participants before the treatment began. Another study’s intervention was implemented for the duration of 12 weeks, and cognitive function was last tested 1 year after the treatment began [58]. Although some studies reported change scores, these measures were not common enough across the full set of studies to use them in our meta-analysis. We conducted the analysis accordingly and used direct post-intervention data when available. One study provided only data that was collected 1 year after the baseline assessment, 9 months after the intervention had ended [58]. Another study had implemented a cognitive sub-study during the follow-up period of the parent study, thereby not providing direct post-intervention data but assessing changes after 1 year [53]. One study reported standard errors instead of standard deviations [56]. We used the reported data in this study to calculate the standard deviation and used our results in the meta-analysis. Details on the reporting of the outcome data can also be found in Table 3. Methodological studies have reported that using post-intervention data will yield, on average, the same results as using change scores when analyzing RCTs [76, 77]. Because this review includes only randomized controlled trials, we considered this approach to be appropriate. The Review Manager 5 provides an estimate of effect sizes computed as Cohen’s *d*. The effect size serves as a quantitative orientation concerning the difference between the intervention (here: vitamins) and control (here: placebo) group in the primary outcome—in our case global cognition. This meta-analysis is depicted in Figs. 4 and 5, including a post hoc subgroup analysis for the defined population group “other risk factors.” The first version of our meta-analysis did not yield a significant effect (*Z* = 0.87; *p* = 0.039; SMD, 0.02; 95% CI, − 0.034, 0.08). The standardized mean difference (SMD) is an indicator for differences in the effects of intervention and placebo [78]. A SMD of 0.02 shows that the effects of vitamin B and placebo are almost equivalent. But, given the measure of heterogeneity, *I*^2^ was 44% and was therefore significant; *p* could not be considered interpretable. Schroll et al. recommend investigating the reasons for significant heterogeneity [79]. Thus, we explored the reasons retrospectively. First, we used the fixed effects model as well as the random effects model and compared the results to evaluate differences with high heterogeneity being present [76, 80]. Suggesting that estimates are related to standard errors, a fixed-effects model is applicable only when effect estimates are not pulled toward the findings of smaller studies [81]. As anticipated, neither the *I*^2^ value nor the results changed significantly. Searching for other reasons, however, we identified one trial that differed from the others in terms of reported data and risk of bias and thereby seemed to affect the overall effect and appeared to be the substantial cause for the significant heterogeneity. This conclusion was supported by a visual examination of the funnel plot (Fig. 5) The majority of studies fell inside the triangular zone in which 95% of studies should be expected to fall when neither bias and heterogeneity are present [81]. However, the deviant trial differed significantly from the others, causing funnel plot asymmetry. Taking into account a visual inspection of the forest plot, the said deviant trial was the only study not meeting the line of null effect, which further strengthens our findings.

**Fig. 6:**
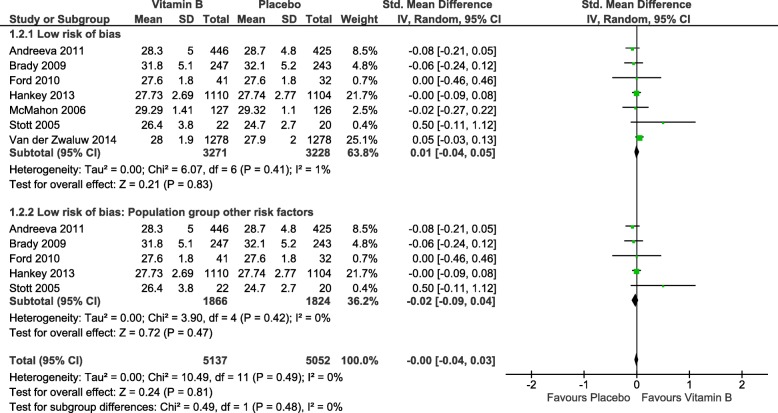
Forest plot: global cognition—sensitivity analysis. SD, standard deviation; CI, confidence interval

**Fig. 7:**
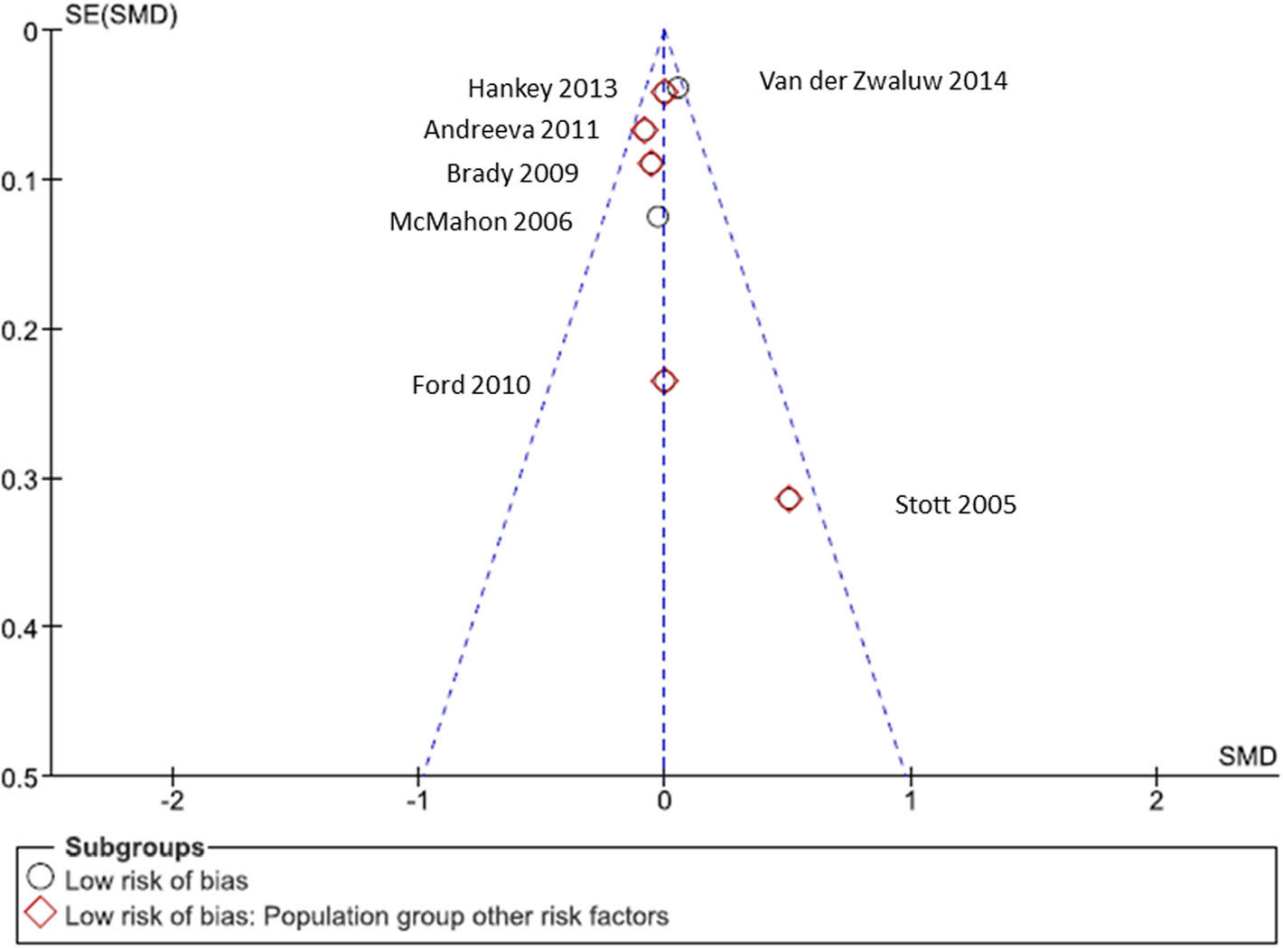
Funnel plot: global cognition—sensitivity analysis

As a consequence, we conducted a sensitivity analysis to exclude studies with two or more high risk of bias ratings. This sensitivity analysis affected only one study that we assumed was causing the high heterogeneity. In the process, the previously significant high heterogeneity was altered to *I*^2^ = 0% (see Fig. 6). This analysis did not yield any significant effect of the intervention displayed in potential differences between the intervention and control groups (*Z* = 0.24; *p* = 0.81; SMD, 0.00; 95% CI, − 0.04, 0.03). An examination of the affiliated funnel plot (presented as Fig. 7) showed that the sensitivity analysis eliminated the funnel plot asymmetry.

In addition, no differences between the intervention and control groups were uncovered by the subgroup analysis, which was conducted on a more homogeneous sample according to the defined population groups.

#### Secondary outcomes

Because of the large variety of outcome measures used in the included studies, the secondary outcomes were displayed according to the four population groups we identified (Table 4). The groups were assessed independently concerning the effects in all reported cognitive domains. This approach was also appropriate for revealing whether significant effects were limited to a specific population group. The cognitive domains we explored and the statistical significance between the control and intervention groups in each trial are presented independently for each population group in the Additional file 3.

In total, potential effects in 28 specific domains were explored and reported. Across the 20 studies, the domains overlapped or were closely related to one another, but they could not be reduced to a common denominator. For example, the construct “memory” alone was depicted in 10 different forms. Because only a few of the reported effects of vitamin B supplementation were significant, we constructed an overview table to demonstrate these significant effects in relation to the population groups. Table 5 shows that 6 out of the 20 studies reported significant effects in cognitive domains other than overall cognition [39, 40, 46, 48, 49, 54]. These domains comprised processing speed, memory (verbal memory), verbal ability, executive function, and attention. However, Table 5 presents only an excerpt of all the potential effects explored in the studies, the majority of which were nonsignificant. The distribution of significant effects was balanced across population groups. Thus, we could not conclude that a specific population group would profit more from the intervention than the other groups. The studies that reported significant effects for secondary outcomes also differed from each other with regard to the numbers of participants (i.e., ranging from 115 to 818 participants), gender restrictions (2 studies assessed women [39, 46], one assessed men [54], and 3 assessed both [40, 48, 49]), intervention details (i.e., different vitamin B administered and differences in the dosage of administered vitamin B), and risk of bias attribution (2 studies were attributed an overall low risk of bias [49, 54], one study was rated with a low to unclear risk of bias [48], 2 studies had an unclear risk of bias [39, 46], and one study had an overall high risk of bias [40]). The distribution of significant effects was not related to any of these differences. All 6 studies assessed elderly individuals (i.e., over 70 years, over 75 years, an age span from 50 to 80 years, and an age span from 60 to 91 years). However, 12 of the 20 studies included in this review administered B vitamins to individuals 60 years of age or older, while only few of the reported cognitive tests in these studies showed significant effects. Overall, none of the 6 studies that reported significant effects showed any particularities that could explain why they achieved significant results for single cognitive domains while the majority of all studies reported nonsignificant results. Due to the high heterogeneity in study characteristics throughout the 6 studies, no specific pattern was observable as to what differentiated these studies from the rest of the studies we included in the systematic review. We found no satisfactory explanation why specifically these 6 studies were able to report significant results while the other 14 studies were not.

**Table 5:**
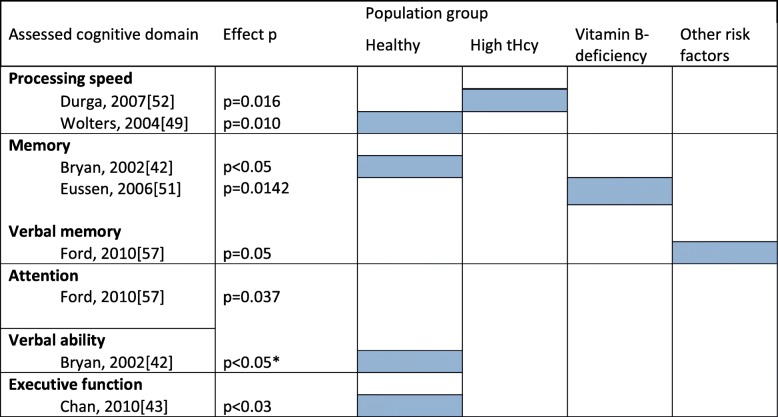
Reported significant effects of secondary outcomes after qualitative synthesis according to population group [39–57]

*p* = *p* value

*This significant effect was reported in favor of the vitamin B_6_ group and the placebo group over the vitamin B_12_ group and folate group

### Risk of bias across studies

We worked with the GRADE approach to assess the risk of bias across studies that could potentially affect the cumulative evidence [34]. The categories included study limitations (risk of bias), publication bias, imprecision, inconsistency, and indirectness. These categories were then cumulated into the overall certainty of evidence. A GRADE analysis was carried out for the studies to which we applied quantitative analyses to assess the primary outcome as well as for the studies that reported significant results for secondary outcomes and for the additional analysis for which the effects favored placebo. The summarized results are presented in Table 6, whereas additional detailed information about how we rated the quality of the evidence is enclosed as Additional file 4. Overall, no serious limitations were detected for any of the outcomes, thus indicating that the evidence could be concluded to have a high level of certainty. This conclusion takes into consideration the finding that one study, because it received an overall high risk of bias rating according to the Cochrane Risk of Bias Tool, was excluded in the second step of the meta-analysis.

**Table 6:** GRADE analysis: summary of findings

Number of participants (studies)	Study limitations (risk of bias)	Publication bias	Imprecision	Inconsistency	Indirectness	Overall certainty of evidence
**Main outcome: overall cognition (measured with TICS-m and MMSE)**						
14.250 (7 RCTs)	No serious limitations	No serious limitations	No serious limitations	No serious limitations	No serious limitations	**++++** High
**Secondary outcome: processing speed**						
1.038 (2 RCTs)	No serious limitations	No serious limitations	No serious limitations	No serious limitations	No serious limitations	**++++** High
**Secondary outcome: memory**						
705 (3 RCTs)	No serious limitations	No serious limitations	No serious limitations	No serious limitations	No serious limitations	**++++** High
**Secondary outcome: attention**						
299 (1 RCT)	No serious limitations	No serious limitations	No serious limitations	No serious limitations	No serious limitations	**++++** High
**Secondary outcome: verbal ability**						
211 (1 RCT)	No serious limitations	No serious limitations	No serious limitations	No serious limitations	No serious limitations	**++++** High
**Secondary outcome: executive function**						
115 (1 RCT)	Serious limitations	No serious limitations	No serious limitations	No serious limitations	No serious limitations	**+++** Moderate
**Additional analysis: effects in favor of placebo**						
610 (3 RCTs)	Moderate limitations	Moderate limitations	No serious limitations	No serious limitations	No serious limitations	**+++** Moderate

### Additional analysis

For safety measures, we conducted an additional analysis to assess the effects in which placebo was favored over vitamin B for the secondary outcomes. For the primary outcome, this was automatically included in the quantitative analyses and can be seen in Figs. 4 and 6. Concerning the secondary outcomes, there were single findings in 4 out of 28 cognitive domains in 3 out of 20 studies that showed a significant effect that favored the placebo [39, 42, 48]. These findings are enclosed as Additional file 5.

## Discussion

### Summary of evidence

In this systematic review, we included 20 RCTs and examined whether a preventive effect of vitamin B on cognitive decline could be observed. The analyses conducted in this review consisted of characteristics of the population groups and interventions, a risk of bias assessment on the study level as well as on the outcome level, a meta-analysis comprising the primary outcome, a subgroup analysis, and a sensitivity analysis. Thus, results for the primary outcome were explored and summarized quantitatively. Secondary outcomes were synthesized qualitatively. For safety reasons, we also conducted an additional analysis to explore effects that favored placebo.

The pathogenic correlation between low vitamin B, high homocysteine, and cognitive decline has been of great interest in recent decades, along with the hope that cognitive benefits would result from modifying these risk factors [14–16, 20, 59–62]. However, older reviews and meta-analyses followed a different approach to addressing this issue than we did, for instance, assessing prevention and treatment of cognitive decline in the same article or focusing exclusively on single vitamin B representatives instead of combinations [23, 25, 26, 28, 82]. One meta-analysis followed an approach that was similar to ours by addressing the same primary outcome that we did, that is, global cognition [24]. However, we included several studies that were not assessed in the older meta-analysis which justifies our analysis.

We focused exclusively on prevention trials and thus excluded people with manifest cognitive impairment, including dementia or mild cognitive impairment. Furthermore, we theoretically included four representatives of vitamin B (B_1_, B_6_, B_12_, and folic acid), although in practice, none of the trials that met our inclusion criteria dealt with the supplementation of vitamin B_1_. Third, we structured the results according to population groups. This decision to assess population groups according to different constellations of risk factors for cognitive decline has not been implemented in a systematic review or meta-analysis before. This is despite the presumption that a potential beneficial effect of vitamin B on cognition would most likely not be applicable to everyone regardless of their cognitive risk factors [29].

On the whole, when integrating the results of the single trials that were analyzed for any population group, no significant effect of vitamin B in slowing cognitive decline could be observed with regard to the primary outcome. This finding held for both healthy individuals and people with some type of cognitive risk factors. The quantitative synthesis resulted in a “zero effect” for the primary outcome. Due to the high heterogeneity displayed in the first version of the meta-analysis, it was necessary to investigate the underlying reasons and to avoid prematurely interpreting the results of this analytic step. After identifying the probable reason for the high heterogeneity, we carried out a second meta-analysis to apply a sensitivity analysis, which resulted in satisfactory and plausible heterogeneity markers. This analysis did not yield a significant effect for the primary outcome and did not show differences for any specific population groups in the subgroup analysis.

We found single significant effects reported for isolated cognitive domains as secondary outcomes in the qualitative synthesis. However, these cases presented a clear minority in comparison with the large number of analyzed cognitive domains in the included trials that did not report significant effects. The majority of the cognitive domains explored in the qualitative analysis did not show any results in favor of vitamin B—regardless of the population group. Some trials indicated significant effects in which the placebo was favored over vitamin B concerning particular cognitive domains. These findings were not associated with a specific population group or other study characteristics that distinguished these studies from the ones that did not report significant effects on the secondary outcomes. In considering the problem of multiple testing and its resulting inflation of the alpha error, it is possible that the single effects that were found to favor vitamin B as well as those that were found to favor the placebo are due to chance. Therefore, these findings should be interpreted with caution [83].

## Limitations

This review addressed a precise research question regarding the terms used in the literature search to retrieve studies involving vitamin B of interest. Because we focused solely on prevention trials, we implemented a first version of the search strategy comprising the keyword “prevention.” However, counter-intuitively, most studies could not be retrieved using the term “prevention” in our search, thus impeding the development of a practicable and appropriate search strategy. Consequently, the use of terms such as “dementia” resulted in the retrieval of a multitude of trials that assessed the effects of treating cognitively impaired individuals with vitamin B and therefore did not meet the inclusion criteria. We subsequently replaced these keywords with the terms “cognition” and “cognitive” in the research question. Given that we restricted our search to articles published in English, it is possible that we failed to retrieve all studies that would be eligible for addressing our research question. However, because the majority of high-quality scientific articles are published in English, it is unlikely that we missed any important studies. The full-texts of all identified studies considered eligible were retrieved. A review protocol was submitted to the PROSPERO register prior to the implementation of our data analysis, thus minimizing reporting bias. As in every systematic review or meta-analysis, the so-called file drawer problem cannot be completely overcome, meaning that we might have overlooked or were not able to retrieve studies that have addressed the same research question but achieved “negative” results (i.e., vitamin B had a negative effect on cognitive function) and were therefore not published. In addition to that, we only included studies that reported outcomes of interest. It is possible that we thereby failed to retrieve studies that did measure outcomes of interest to our review but did not report them, e.g., because of nonsignificant results. However, since we already identified a “zero result” with the published studies available to us, suggesting that vitamin B have not been shown to prevent cognitive decline, the impact of this possible bias on our conclusion would be negligible. Furthermore, our funnel plot did not suggest a relevant imbalance in the included studies and did not indicate that a publication bias was likely.

Given our eligibility criteria, this review did not include results for any forms of administration of vitamin B other than oral supplementation. Trials assessing other forms of administration were excluded during the screening process but could nonetheless provide a foundation for further research and subsequent reviews [84, 85]. In our review, we exclusively assessed the preventive possibilities of vitamin B on cognitive decline and can therefore offer no conclusions about potential therapeutic effects for individuals with cognitive impairment.

The characteristics of the included studies limited this review with respect to different parameters. First of all, not all studies assessed changes in cognitive function as the primary outcome of their trials. The assessed population was characterized by a wide age span as well as by a multitude of different risk factors and diseases that potentially lead to cognitive decline. By categorizing these differences into four population groups, we aimed to provide a better distinction between risk factors. Given that we included studies that investigated the supplementation of single and combined vitamin B, the potential beneficial effects cannot be readily ascribed to a definite source, that is, a specific representative of vitamin B. Furthermore, we did not perform subgroup analyses regarding trials that were performed in countries where food fortification with vitamin B is allowed. Taking into account the fact that this review identified no overall significant effect of the intervention, we can consider these limitations negligible. The duration of treatment in the included studies differed from 5 weeks to 6.6 years and thereby led to limited comparability across studies. Again, because no overall beneficial effect could be observed regardless of the duration, these differences are of minor importance. Most studies reported direct post-intervention data concerning the cognitive tests they implemented. Due to the variability in the duration of the interventions, however, comparability was limited here for the same reasons. The included studies reported 86 tests on 28 different cognitive outcomes, thereby impeding the qualitative analysis. For the most part, assessments of cognitive constructs were not comparable throughout the studies due to high levels of heterogeneity in the reported data; the construct “memory” alone was depicted in 10 different forms. Apart from our primary outcome “global cognition,” there was no apparent inclination to use certain favored cognitive constructs. Even when studies focused on similar cognitive domains, they reported a multitude of different tests for assessing the same construct and thereby prohibited the ability to obtain a unified picture.

The included studies varied greatly regarding their case numbers. Small sample sizes impede the obtaining of meaningfully interpretable results while larger studies should add a greater value to the overall results of a systematic review. However, during the course of the implemented meta-analysis, the included studies were automatically weighted on the basis of their case numbers, with larger studies being assigned a greater percentage to the overall result.

Furthermore, only one study aimed to report the conversion of no cognitive impairment to dementia as an example for cognitive impairment [55]. Thus, analyses investigating risk reduction with prevention measures were not possible. The construct “global cognition” was already a less common denominator in the studies we analyzed. Only 8 of the 20 trials in the systematic review assessed this construct [50–56, 58]. Global cognition was additionally limited by the fact that it was assessed with two different procedures (that is MMSE and TICS-m), even though there were strong hints for comparability [36–38]. In addition, there has been legitimate criticism in the scientific community concerning the MMSE for several decades [86]. However, it is still a very common, widely used, and clinically accepted screening instrument [75]. This is also the case of the studies included in this research work, which clearly present with a lack of alternative assessment tools to measure global cognition appropriately. We are aware of the shortcomings the MMSE suffers from and acknowledge the potentially limiting effect this has on our results concerning the primary outcome of this review. Investigations have shown that the MMSE has only limited validity for differentiating persons with mild cognitive impairment from healthy individuals [75, 87, 88]. Since we did not use this cognitive test to diagnose MCI or dementia and only observed changes in measurements in both the intervention and control groups, an interpretation of the results is justifiable in our case. The MMSE has also been proven to be notoriously insensitive to mild cognitive changes [89]. Furthermore, the MMSE only has a limited sensitivity regarding changes in cognitive function over time. Its value in measuring the progression of cognitive decline improves for periods longer than 3 years [90]. This could be a reason for nonsignificant study results, especially in studies with small group samples or a short duration of the intervention. However, several of the studies we comprised in the meta-analysis assessed over 2000 participants. In 7 of the 8 studies we included in the meta-analysis, the intervention continued for at least 2 years [50–56] and over 3 years in 4 of the 8 studies [52, 53, 55, 56]. The last follow-up assessments synchronized with the end of the treatment in 6 out of 8 studies [50–52, 54–56] and were conducted 1 year after the end of the treatment in the other 2 studies [53, 58]. One study had a significantly smaller group sample and a significantly shorter observation period than the others but had only a minor influence on the overall result of the meta-analysis [58]. Thus, we can consider this potential influence on the reported changes in the MMSE scores negligible. There is no logical reason to assume that participants in the control groups could have been more strongly biased than participants in the intervention groups concerning the measurement instrument. We assume that potential measurement problems, (e.g., sensitivity to change), would have affected both groups in the same manner.

For the risk of bias assessment on the study level, we chose a conservative approach, adhering strictly to the Cochrane Risk of Bias guidelines [32]. We further assessed the risk of bias for the reported outcomes by following the GRADE approach to ensure that the quality of evidence was thoroughly evaluated [33–35]. One study to which we attributed a high risk of bias caused significant heterogeneity and funnel plot asymmetry in our first meta-analysis and was thus excluded on the basis of a sensitivity analysis (final model) after we investigated the reasons for heterogeneity and asymmetry [56]. We observed no connection between the ratings in isolated categories of the Cochrane Risk of Bias Tool and specific population or study characteristics.

## Conclusion

On the whole, this review included 20 studies with a variety of characteristics, none of them providing an overall significant effect of vitamin B supplementation on cognitive function. The meta-analysis and sensitivity analysis we conducted did not yield a significant effect when we used a fixed-effects model as well as the more conservative random-effects model which was ultimately used in our meta-analysis. Effects did not differ in the subgroup analysis for the population group comprising other risk factors.

This review is a further addition to several similar articles on the influence of vitamin B on cognitive function. Nevertheless, it is an important topic in the field of nutrition and cognition and hence always worth an updated systematic review and meta-analysis. We aimed for an approach that would distinguish this review from previous articles. Unlike older reviews and meta-analyses, we took into consideration the fact that, despite the absence of cognitive impairment, different risk factors for developing cognitive decline may be present for the participants and therefore clustered the results accordingly. Furthermore, we assessed different studies, especially for our primary outcome. Nevertheless, our results are aligned with the conclusions of older reviews and meta-analyses on this topic [23, 24, 26, 28, 91, 92].

Older studies and reviews have supported a future implication of further long-term RCTs for assessing the potential influence of oral vitamin B supplementation on cognitive decline [91, 93, 94]. Independent from the length of the intervention or the study, we found no hint that B vitamin supplementation offered a positive effect on “global cognition,” the primary outcome of this review and meta-analysis. Moreover, the studies we included in the review did not report an overall beneficial effect regardless of not only the duration of the treatment but also the characteristics of the population (including among others gender and age characteristics), indicating that this approach might not be suitable for reliably preventing cognitive decline after all.

We found no evidence to support the hypothesis that one of the assessed population groups would profit more than others from vitamin B with respect to cognitive function. Why certain studies were able to report isolated significant effects of vitamin B on cognition while the majority of the effects reported were nonsignificant has not been finally clarified over the course of this systematic review. This includes the effects that favored the treatment as well as those that favored the placebo.

Consequently, we cannot impartially recommend the use of oral vitamin B to prevent cognitive decline in cognitively unimpaired individuals without further clarification of its influence on the specific cognitive domains that we included as secondary outcomes and that reported significant effects.

However, this conclusion can be drawn only for the oral supplementation of vitamin B and does not apply to any other form of administration of vitamin B. It is furthermore only applicable to cognitively unimpaired individuals and does not allow conclusions to be drawn for individuals with cognitive impairment.

Because this review found no evidence that vitamin B supplementation causes harm to cognitive function, further research in which different aspects of the potential health benefits of vitamin B are studied can be supported. The connection between vitamin B, homocysteine, and cognition is a much studied and promising field and should be further addressed in future high-quality trials. Further clarification regarding the influence of vitamin B on the isolated cognitive domains with reported significant effects in this review is needed. However, in the context of this review, we recommend that researchers also concentrate on different aspects, such as other forms of administering vitamin B as well as their influence on cognitively impaired individuals. The influence of vitamin B supplementation on diagnosed MCI is another crucial topic in this field of research. Several trials that were excluded in the screening process for eligibility for this review offer promising results regarding the treatment of MCI via vitamin B, underlining the need for further investigation [95–98]. An issue addressed in this review as well as in older reviews was the amount and heterogeneity of cognitive assessment tools used across the studies [91, 99]. To ensure better comparability in the reporting of outcomes between studies, future research should preferably implement fewer and well-established cognitive tests with high sensitivity to change in their studies. More specific assessment tools could instead be included in supplementary analyses.

The results from this review are relevant for providers in the health sector and for policy makers, because the oral supplementation of vitamin B to prevent cognitive decline does not seem to offer a profitable investment. Second, physicians, especially general practitioners should be able to provide well-founded knowledge on potential preventive approaches to their patients and could therefore profit from this review. Ultimately, our findings are of general public interest and are relevant for each individual because everyone must be given the opportunity to inform themselves about which approaches might be able to prevent cognitive impairment and which might not. In our case, we have to conclude that one cannot expect to prevent cognitive decline by taking oral vitamin B supplementation when there is no cognitive impairment at the beginning of the supplementation. On the other hand, one has no reason to anticipate serious inverse effects from such an intervention either.

## Supplementary information

**Additional file 1. **PRISMA 2009 Checklist.

**Additional file 2.** PICOS eligibility criteria as defined before initial database search.

**Additional file 3.** Detailed overview of secondary outcomes according to population groups (A-D).

**Additional file 4.** Detailed GRADE analysis for the primary and the secondary outcomes (A-E).

**Additional file 5.** Significant effects of secondary outcomes in favor of placebo.

## Data Availability

All data generated or analyzed in this systematic review and meta-analysis are included in this published article and its supplementary information files.

## Abbreviations

Vitamin B: Vitamin B_1_, B_6_, B_12_, and folic acid
MCI: Mild cognitive impairment
MMSE: Mini-Mental State Examination
TICS-m: Telephone Interview for Cognitive Status
RCT: Randomized controlled trial
CI: Confidence interval
SD: Standard deviation
ES: Effect size
*p*: *p* value
