# Supplementary material for: Vitamin B—Can it prevent cognitive decline? A systematic review and meta-analysis
Source: Syst Rev. 2020 May 15;9:111. doi: 10.1186/s13643-020-01378-7 (PMC7229605; doi:10.1186/s13643-020-01378-7)
Supplement: Supplementary file 2 — Additional file 2. PICOS eligibility criteria as defined before initial database search. [file 13643_2020_1378_MOESM2_ESM.docx]

**Additional file 2. PICOS eligibility criteria as defined before initial database search.**

|  | Included in screening process | Excluded from screening process |
| --- | --- | --- |
| Patient population | Healthy individuals  Pre-existing conditions that constitute potential risk factors for cognitive impairment | Any form of cognitive impairment  Pre-existing mental disorders  Children and adolescents  Pregnant women |
| Intervention | Oral supplementation with Vitamin B | Subcutaneous application of Vitamin B |
| Comparison | Control group (placebo) | Other substances or interventions |
| Outcome | Cognitive performance | Non-cognitive outcome measures |
| Study design | RCTs  Controlled studies | Reviews  Commentaries  Non-controlled studies (pre-post design) |
