# Supplementary material for: Vitamin B—Can it prevent cognitive decline? A systematic review and meta-analysis
Source: Syst Rev. 2020 May 15;9:111. doi: 10.1186/s13643-020-01378-7 (PMC7229605; doi:10.1186/s13643-020-01378-7)
Supplement: Supplementary file 3 — Additional file 3. Detailed overview of secondary outcomes according to population groups (A-D). [file 13643_2020_1378_MOESM3_ESM.docx]

| **Additional file 3. Detailed Overview of Secondary Outcomes According to Population.** | | | | | | | | | | |
| --- | --- | --- | --- | --- | --- | --- | --- | --- | --- | --- |
| **A. Population group: Healthy** | | | | | | | | | | |
| **Authors** | **Memory** | **Percep-tual speed** | **Verbal ability** | **Spatial ability** | **Reasoning** | **Attention** | **Processing speed** | **Executive function** | **Integrative capacity** | **Sensori-motor speed** |
| Bryan, 2002 | Overall memory:  p<0.05 (in favor of older age in folate group) |  | p<0.05 (in favor of Vitamin B6 group and placebo) |  |  |  | No significant  difference  (data not  shown) | No significant difference (data not shown) |  |  |
|  | Working memory:  No significant difference (data not shown) |  |  |  |  |  |  |  |  |  |
| Chan, 2010 | Verbal memory: p=0.35 (overall composite |  |  |  |  |  |  | p<0.03 |  |  |
| Cockle, 2000 | Short term memory, long term memory, recognition memory:  No significant difference (data not shown) |  | No significant difference (data not shown) |  |  |  |  |  | No overall significant difference (data not shown) | No significant difference (data not shown) |
| Lewerin, 2004 | Short term memory, visual memory:  No significant difference (data not shown)  Long term memory:  p=0.197 | p=0.039 p=0.093 | p=0.017 (in favor of placebo) | No significant difference (data not shown) | p=0.164 (in favor of placebo) |  |  |  |  |  |
| Pathansali, 2006 | Memory, visuo-spatial memory, associative memory:  No significant difference |  |  |  | No significant  difference | No significant  difference |  |  |  |  |
| Pipingas, 2013 | Working memory:  p=0.10  Recognition memory:  p=0.91  p=0.14  p=028 |  |  |  |  | p=0.9 p=0.75 p=0.09 p=0.22 p=0.96 | p=0.9 p=0.75 p=0.09 p=0.22 p=0.96 |  |  |  |
| Walker, 2012 |  |  |  |  |  |  | No significant difference (data not shown) |  |  |  |
| Wolters, 2004 |  |  |  |  |  |  | p=0.010 |  |  |  |

| **B. Population group: Vitamin B-deficiency** | | | | | | | |
| --- | --- | --- | --- | --- | --- | --- | --- |
| **Authors** | **Memory** | **Processing speed** | **Reaction time** | **Executive function** | **Attention** | **Construction** | **Sensorimotor speed** |
| Dangour, 2015 | no significant difference (data not shown) | no significant difference (data not shown) | no significant difference (data not shown) | no significant difference (data not shown) |  |  |  |
| Eussen, 2006 | p=0.0142 |  |  | no significant difference (data not shown) | no significant difference (data not shown) | no significant difference (data not shown) | no significant difference (data not shown) |

| **C. Population group: High homocysteine levels** | | | | | | | | | |
| --- | --- | --- | --- | --- | --- | --- | --- | --- | --- |
| **Authors** | **Memory** | **Attention** | **Sensorimotor speed** | **Complex speed** | **Processing speed** | **Executive function** | **Word fluency** | **Semantic fluency** | **Reasoning ability** |
| Durga, 2007 | Overall memory:  p=0.01 |  | p=0.055 | p=0.4 | p=0.016 |  | p=0.245 |  |  |
| McMahon, 2006 | Overall memory:  p=0.12 p=0.14 p=0.16 (all in favor of placebo) |  |  |  | p=0.007 (in favor of placebo) |  | p=0.74 (in favor of placebo) | p=0.48 (in favor of placebo) | p=0.22 (in favor of placebo) |
| Van der Zwaluw, 2014 | Episodic memory:  p=0.42  Working memory:  No significant difference (data not shown) | No significant difference (data not shown) |  |  | no significant difference (data not shown) | no significant difference (data not shown) |  |  |  |

| **D. Population group: Other risk factors** | | | | | | | |
| --- | --- | --- | --- | --- | --- | --- | --- |
| **Authors** | **Memory** | **Attention** | **Executive function** | **Visuospatial skills** | **Planning** | **Information processing speed** | **CDR (Clinical Dementia Rating)** |
| Brady, 2009 | Working memory:  p=0.3 | p=0.8 | p=0.2 |  |  |  |  |
| Ford, 2010 | Verbal memory:  p=0.05 | p=0.037 |  | no significant difference (data not shown) | no significant difference (data not shown) |  |  |
| Kang, 2008 |  | no significant difference (data not shown) | no significant difference (data not shown) |  |  |  |  |
| Kwok, 2016 |  |  |  |  |  |  | no significant difference (data not shown) |
| Stott, 2005 |  | no significant difference (data not shown) |  |  |  | no significant difference (data not shown) |  |
