# Supplementary material for: Vitamin B—Can it prevent cognitive decline? A systematic review and meta-analysis
Source: Syst Rev. 2020 May 15;9:111. doi: 10.1186/s13643-020-01378-7 (PMC7229605; doi:10.1186/s13643-020-01378-7)
Supplement: Supplementary file 4 — Additional file 4. Detailed GRADE analysis for the primary and the secondary outcomes (A-E). [file 13643_2020_1378_MOESM4_ESM.docx]

**Additional file 4. Detailed GRADE analysis for the primary and the secondary outcomes (A-E).**

| **A. Primary outcome: Overall cognition** | | | | | | |
| --- | --- | --- | --- | --- | --- | --- |
| **Study** | **Number of participants (study total)*** | **Study limitations (risk of bias)** | **Publication bias** | **Imprecision** | **Inconsistency** | **Indirectness** |
| Andreeva, 2011 | 1748 | Unclear | Not industry-sponsored, no small study | Confidence Interval 95% |  |  |
| Brady, 2009 | 659 | Low | Not industry-sposored, no small study | Conficence Interval 95% |  |  |
| Ford, 2010 | 299 | Low | Not industry-sponsored, rather small study | Confidence Interval 95% |  |  |
| Hankey, 2013 | 8164 | Low | Not industry-sponsored, no small study | Confidence Interval 95% |  |  |
| Kang, 2008 | 2009 | High | Not industry-sponsored, no small study | Confidence Interval 95% |  |  |
| McMahon, 2006 | 276 | Low | Not industry-sponsored, rather small study | Confidence Interval 95% |  |  |
| Stott, 2005 | 185 | Low | Not industry-sponsored,small sample size | Confidence Interval 95% |  |  |
| Van der Zwaluw, 2014 | 2919 | Low | Not industry-sponsored, no small study | Confidence Interval 95% |  |  |
|  | 14.250 | No serious limitation  One study out of 7 was rated unclear instead of low risk of bias. One study was rated high risk of bias but was excluded in the second analysis step of the meta-analysis (sensitivity analysis). | No serious limitation  Only one study had a small sample size. | No serious limitation | No serious limitation | No serious limitation |

*Total of study participants, which does not necessarily represent the number of participants tested for this outcome. (The study’s total number of participants is important regarding the rating of publication bias (↗GRADE guidelines: 5. Rating the quality of evidenced publication bias)

| **B. Secondary outcome: Pprocessing speed** | | | | | | |
| --- | --- | --- | --- | --- | --- | --- |
| **Study** | **Number of participants (study total)*** | **Study limitations (risk of bias)** | **Publication bias** | **Imprecision** | **Inconsistency** | **Indirectness** |
| Durga, 2007 | 818 | Low | Not industry-sponsored, no small study | Confidence Interval 95% |  |  |
| Wolters, 2004 | 220 | Unclear | Not industry-sponsored, rather small study | Confidence Interval 95% |  |  |
|  | 1.038 | Unlikely to lower confidence in the estimate of effect.  →No serious limitation | No serious limitation | No serious limitation | No serious limitation | No serious limitation |

*Total of study participants, which does not necessarily represent the number of participants tested for this outcome. (The study’s total number of participants is important regarding the rating of publication bias (↗GRADE guidelines: 5. Rating the quality of evidenced publication bias)

| **C. Secondary outcome:Memory** | | | | | | |
| --- | --- | --- | --- | --- | --- | --- |
| **Study** | **Number of participants (study total)*** | **Study limitations (risk of bias)** | **Publication bias** | **Imprecision** | **Inconsistency** | **Indirectness** |
| Bryan,  2002 | 211 | Unclear | Not industry-sponsored, rather small study | Confidence Interval 95% |  |  |
| Eussen, 2006 | 195 | Unclear | Partially industry-sponsored, small sample size | Confidence Interval 95% |  |  |
| Ford, 2010 | 299 | Low | Not industry-sponsored, rather small study | Confidence Interval 95% |  |  |
|  | 494 | No serious limitation  Unlikely to lower confidence in the estimate of effect. | No serious limitation | No serious limitation | No serious limitation | No serious limitation |

*Total of study participants, which does not necessarily represent the number of participants tested for this outcome. (The study’s total number of participants is important regarding the rating of publication bias (↗GRADE guidelines: 5. Rating the quality of evidenced publication bias)

| **D. Secondary outcome:Attention** | | | | | | |
| --- | --- | --- | --- | --- | --- | --- |
| **Study** | **Number of participants (study total)*** | **Study limitations (risk of bias)** | **Publication bias** | **Imprecision** | **Inconsistency** | **Indirectness** |
| Ford, 2010 | 299 | Low | Not industry-sponsored, rather small study | Confidence Interval 95% |  |  |
|  | 299 | No serious limitation | No serious limitation | No serious limitation | No serious limitation | No serious limitation |

*Total of study participants, which does not necessarily represent the number of participants tested for this outcome. (The study’s total number of participants is important regarding the rating of publication bias (↗GRADE guidelines: 5. Rating the quality of evidenced publication bias)

| **D. Secondary outcome : Verbal ability** | | | | | | |
| --- | --- | --- | --- | --- | --- | --- |
| **Study** | **Number of participants (study total)*** | **Study limitations (risk of bias)** | **Publication bias** | **Imprecision** | **Inconsistency** | **Indirectness** |
| Bryan, 2002 | 211 | Unclear | Not industry-sponsored, rather small study | Confidence Interval 95% |  |  |
|  | 211 | No serious limitation | No serious limitation | No serious limitation | No serious limitation | No serious limitation |

*Total of study participants, which does not necessarily represent the number of participants tested for this outcome. (The study’s total number of participants is important regarding the rating of publication bias (↗GRADE guidelines: 5. Rating the quality of evidenced publication bias)

| **D. Secondary outcome: Executive function** | | | | | | |
| --- | --- | --- | --- | --- | --- | --- |
| **Study** | **Number of participants (study total)*** | **Study limitations (risk of bias)** | **Publication bias** | **Imprecision** | **Inconsistency** | **Indirectness** |
| Chan, 2010 | 115 | High | Not industry-sponsored, rather small study | Confidence Interval 95% |  |  |
|  | 115 | Serious limitation  Potentially lowering confidence in the estimate of effect | No serious limitation | No serious limitation | No serious limitation | No serious limitation |

*Total of study participants, which does not necessarily represent the number of participants tested for this outcome. (The study’s total number of participants is important regarding the rating of publication bias (↗GRADE guidelines: 5. Rating the quality of evidenced publication bias)

| **E. Secondary outcome “effects in favor of placebo”** | | | | | | |
| --- | --- | --- | --- | --- | --- | --- |
| **Study** | **Number of participants (study total)*** | **Study limitations (risk of bias)** | **Publication bias** | **Imprecision** | **Inconsistency** | **Indirectness** |
| Eussen et al, 2006 | 195 | Unclear | -Partially industry-sponsored, mall sample size | Confidence Interval 95% |  |  |
| Lewerin et al, 2005 | 195 | Moderate | Not industry-sponsored, small sample size | Confidence Interval 95% |  |  |
|  | 390 | Moderate limitation  Potentially lowering confidence in the estimate of effect. | Moderate limitation | No serious limitation | No serious limitation | No serious limitation |

*Total of study participants, which does not necessarily represent the number of participants tested for this outcome. (The study’s total number of participants is important regarding the rating of publication bias (↗GRADE guidelines: 5. Rating the quality of evidenced publication bias)
