# Supplementary material for: Vitamin B—Can it prevent cognitive decline? A systematic review and meta-analysis
Source: Syst Rev. 2020 May 15;9:111. doi: 10.1186/s13643-020-01378-7 (PMC7229605; doi:10.1186/s13643-020-01378-7)
Supplement: Supplementary file 5 — Additional file 5. Significant effects of secondary outcomes in favor of placebo. [file 13643_2020_1378_MOESM5_ESM.docx]

| **Additional file 5. Significant effects of secondary outcomes in favor of placebo.** | | | | | | |
| --- | --- | --- | --- | --- | --- | --- |
| **Study** | **Cognitive domain** | **Effect (p)** | **Population group** | | | |
|  |  |  | **Healthy** | **High tHcy** | **Vitamin B-deficiency** | **Other risk factors** |
| Eussen et al., 2006 | Memory | p=0.0036 |  |  |  |  |
| Lewerin et al., 2005 | Identical Forms | p=0.039 |  |  |  |  |
|  | Synonyms | p=0.017 |  |  |  |  |
| Bryan et al., 2002 | Verbal ability | p<0.05 * |  |  |  |  |

*This significant effect was reported in favor of the Vitamin B6 group and the placebo group over the Vitamin B12 group and folate group.
